# The Economic Value of Antimicrobial Use in Livestock Production

**DOI:** 10.3390/antibiotics12101537

**Published:** 2023-10-13

**Authors:** Jamal L. Roskam, Alfons G. J. M. Oude Lansink, Helmut W. Saatkamp

**Affiliations:** Business Economics Group, Wageningen University & Research, Hollandseweg 1, 6706 KN Wageningen, The Netherlands

**Keywords:** antibiotic use, antimicrobial use, economic value

## Abstract

(1) Introduction: Antimicrobial agents have played an important role in improving the productivity of worldwide livestock production by reducing the impact of livestock diseases. However, a major drawback of antimicrobial use is the emergence of antimicrobial-resistant pathogens in food-producing animals. To reduce the use of antimicrobials, it is important to know the economic value of the use of antimicrobials and factors that determine that economic value. (2) Results: A theoretical framework was developed to assess the economic value of antimicrobial use. Three situations were distinguished: firstly, a baseline model for a farm with a conventional production system; secondly, an extension of the baseline model that includes the impact of production system improvements; and thirdly, an extension of the baseline model that includes the impacts of risk and risk attitude. This framework shows that the economic value of antimicrobial use is negatively affected by the price of productive inputs and damage-abatement inputs, and positively affected by the output price, the input–output combination, the damage abatement effect, risk aversion and variance in profit. (3) Conclusions: The theoretical framework presented in this study shows that there are several factors that (can) affect the economic value of antimicrobial use. The knowledge about the effect of these factors can be utilized to affect the economic value of antimicrobials and, consequently, affect antimicrobial use.

## 1. Introduction

Antimicrobial agents (AMs) have played an important role in improving the productivity of worldwide livestock production by reducing the impact of livestock diseases. Various purposes of antimicrobial use (AMU) are distinguished [1,2,3,4]: therapeutic purposes (i.e., treatment of diseased animals), prophylactic purposes (i.e., disease prevention), metaphylactic purposes (i.e., administration to clinically healthy animals that belong to a herd or flock with clinical signs) and growth promotion. A major drawback of AMU is the emergence of antimicrobial-resistant pathogens in food-producing animals. Contamination of food products with resistant pathogens can cause antimicrobial resistance (AMR), which could reduce antimicrobial effectiveness in humans [5]. AMR is, therefore, considered a major global health threat [6,7].

Various studies have provided evidence of a relation between AMU in livestock production and the prevalence of resistant pathogens in humans [8,9,10,11,12,13,14,15]. Hence, there is an urgent need for reducing AMU in livestock production to a minimum required to guarantee animal health but still be compatible with sustainable animal production [16,17,18,19,20]. Currently, there is no agreement about what this minimum should be. Different measures have contributed to reductions in AMU, including a European ban in 2006 on the use of antimicrobial growth promoters. 

Successful measures have led to a significant reduction in veterinary AMU in countries such as the Netherlands and Denmark. However, large variations remain in AMU between countries and individual farms. This suggests possibilities for further reductions in AMU, in particular among farmers with intensive AMU. Individual farmers should, therefore, be at the core of any effort to reduce AMU. Hence, an understanding of the economic value of antimicrobial use (EV_AMU_) and the factors that affect the EV_AMU_ is essential, and this knowledge can be utilized to derive concrete policy recommendations from future empirical studies to affect the EV_AMU_ in order to reduce AMU.

The objective of this study was twofold. First, this study developed a theoretical framework to derive the EV_AMU_. Second, this study utilized the developed framework to determine the factors that affect the EV_AMU_, which can be used to reduce AMU in livestock production. The emphasis of the theoretical framework presented in this study is on intensive livestock production systems.

## 2. Results

The production function expresses the technical relationship between inputs used and outputs produced [21,22]. This section provides different production function specifications that explicitly account for the role of damage-abatement inputs. Damage-control agents do not enhance productivity directly, in contrast to production factors known as productive inputs [23]. Hence, damage-abatement inputs are defined as inputs that reduce damage rather than increase the output, whereas productive inputs are inputs that increase the output directly [24]. The traditional specification of production function is as follows:

(1) $$y=F\left(x,z\right)$$

where x is a vector of productive inputs and z is a vector of damage-abatement inputs. $F\left(\cdot \right)$ is assumed to possess standard production function properties, in particular concavity in $\left(x,z\right)$. Hence, the traditional production function specification, for example, the specification used to show how animal diseases could alter the shape of the function [25], treats x and z symmetrically.

Damage-abatement specifications differ from the traditional specification in the asymmetric treatment of productive inputs and damage-abatement inputs. The concept of damage-abatement inputs was introduced to the agricultural economics literature by Hall and Norgaard [26] and Talpaz and Borosh [27]. An output damage abatement production function was specified by Lichtenberg and Zilberman [23], which is consistent with the concept of damage abatement and allows damage-abatement inputs to reduce losses from potential output. Following Lichtenberg and Zilberman [23], the specification of the output damage abatement production function is as follows:

(2) $$y=F\left(x\right)\cdot D\left(z\right)$$

where $F\left(\cdot \right)$ is a production function that gives the potential output $\left(y\right)$ from the productive input vector x, and $D\left(\cdot \right)$ is a damage abatement function that gives the level of damage abatement from the damage-abatement input vector z. Following Oude Lansink and Carpentier [24], the properties of the output damage abatement production function are as follows:

(3a) $$D\left(z\right)\geq 0$$

(3b) $$0\leq D\left(z\right)\leq 1$$

Property $\left(3a\right)$ implies that damage-abatement inputs are not strictly essential inputs, i.e., positive damage abatement is possible at the zero levels of damage-abatement inputs. Property $\left(3b\right)$ implies that the damage abatement function is defined as a fraction between zero and one. $D\left(z\right)=1$ indicates that the destructive capacity is completely eliminated, i.e., losses are zero and the actual output equals the potential output [23]. In practice, $D\left(z\right)$ could never be equal to one because of an inevitable damage if antimicrobial treatments are applied after the appearance of disease effects. $D\left(z\right)=0$ denotes the output obtainable under maximum destructive capacity, i.e., the actual output equals the minimum output [23].

The theoretical framework proposed in this study follows the output damage abatement production function specification of Lichtenberg and Zilberman [23]. This specification assumes that no interdependence exists between productive inputs and damage-abatement inputs. An alternative specification was introduced by Oude Lansink and Carpentier [24] to capture the potential interdependence between productive inputs and damage-abatement inputs, i.e., damage-abatement inputs affect the productivity of productive inputs. However, this assumption is arguable in the case of AMU as the effect of AMU on the productivity of productive inputs (including feed) is similar to growth promotion, which is prohibited in the EU.

The theoretical framework presented in this study assesses the ${\mathrm{EV}}_{\mathrm{AMU}}$ for individual farmers in three specific situations: (1) a baseline model that examines the ${\mathrm{EV}}_{\mathrm{AMU}}$ for a specific farm with a conventional production system, (2) an extension of the baseline model that includes the impact of production system improvements, and (3) another extension of the baseline model that includes the impacts of risk and risk attitude.

### 2.1. Baseline Model

**Figure 1:**
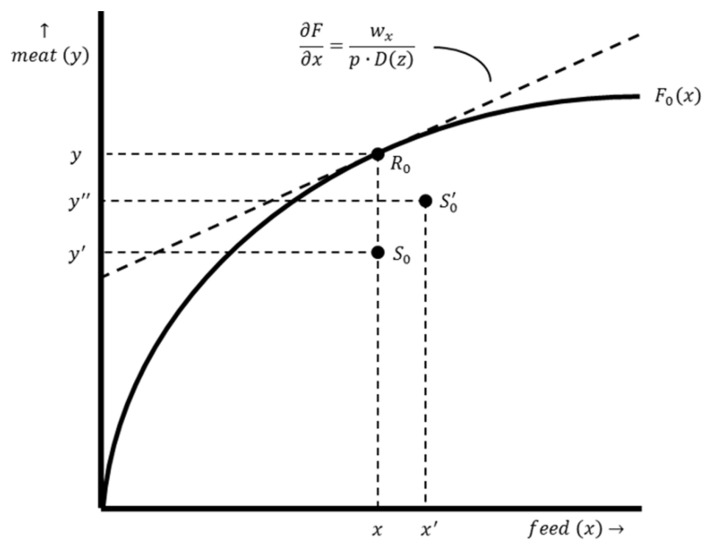
The production function of a farm with a conventional production system. Production function $F_{0}\left(x\right)$ illustrates the relation between the output, meat $\left(y\right),$ and the productive input, feed $\left(x\right),$ for a specific farm with a conventional production system. The dashed line represents the isoprofit line. The slope of this line is $\frac{\partial F}{\partial x}=\frac{w_{x}}{p\cdot D\left(z\right)}$. Point $R_{0}$ shows the optimal input–output combination $\left(x,y\right)$ for a production level without damage. Point $S_{0}$ shows the input–output combination $\left(x,y^{\prime }\right)$ with maximum damage and no AMU. Point $S_{0}^{\prime }$ shows the input–out combination $\left(x^{\prime },y^{\prime \prime }\right)$ resulting from optimal damage abatement.

The starting point of the baseline model is the production function, which hypothetically expresses the relationship between the output, meat (*y*-axis), and the main productive input, feed (*x*-axis) (see Figure 1). In practice, the relationship between the output and the productive inputs is multidimensional because multiple productive inputs are used to obtain the output. The production function slopes upward because more meat is produced when more feed is used. The marginal product of feed declines when more feed is used, i.e., the marginal product of feed drops as the amount of feed used increases, and therefore, there are diminishing returns for feed [22].

Neoclassical economics is based on a number of assumptions. In the baseline model, farmers are assumed to maximize profit. Let p denote the price of output, $w_{x}$ denote the price of productive inputs, and $w_{z}\;$ denote the price of damage-abatement inputs, then the relevant profit maximization problem is expressed as follows:

(4) $$\underset{x,z}{max}\pi =p\left(F\left(x\right)\cdot D\left(z\right)\right)-w_{x}\cdot x-w_{z}\cdot z$$

According to the conventional profit maximization criterion, the optimal input–output combination is denoted as point $R_{0}$ in Figure 1. At this point, the production function is tangent to the isoprofit line. This involves a tangency condition in which the slope of the production function (i.e., the marginal product of productive input use) equals the slope of the corresponding isoprofit line. This point is found by taking the first derivative of π with respect to x, setting it equal to zero and then rewriting it in terms of ∂F/∂x (see Equations (5) and (6)).

(5) $$\frac{\partial \pi }{\partial x}=p\left(\frac{\partial F}{\partial x}\cdot D\left(z\right)\right)-w_{x}=0$$

(6) $$\frac{\partial F}{\partial x}=\frac{w_{x}}{p\cdot D\left(z\right)}$$

Equations (5) and (6) show that the marginal product of productive input use is determined by $w_{x}$, p and $D\left(z\right)$. A rational farmer starts using more (less) productive inputs when the price of productive inputs decreases (increases) and/or the output price increases (decreases), ceteris paribus (cet. par.). Hence, the marginal product will increase (decrease) when $w_{x}$ decreases (increases), cet. par. In addition, the marginal product will increase (decrease) when p increases (decreases), cet. par. The same effect applies to $D\left(z\right)$, cet. par.

**Figure 2:**
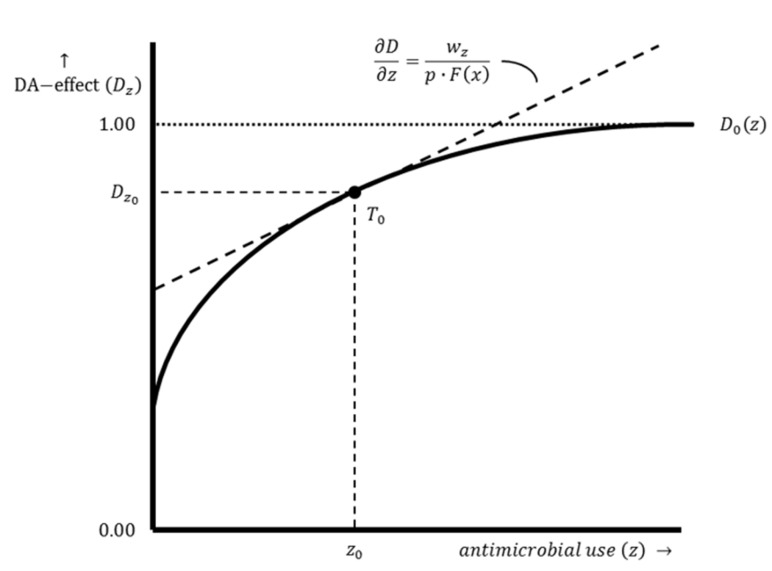
The damage abatement function of a farm with a conventional production system. Damage abatement function $D_{0}\left(z\right)$ illustrates the relation between the damage abatement effect $\left(D_{z}\right)$ and the damage-abatement input use $\left(z\right)$ for a farm with a conventional production system. The dashed line represents the isoprofit line. The slope of this line is $\frac{\partial D}{\partial z}=\frac{w_{z}}{p\cdot F\left(x\right)}$. Point $T_{0}$ shows the optimal damage abatement effect $D_{z_{0}}$ resulting from an optimal level of AMU $\left(z_{0}\right)$.

Point $R_{0}$ is located on the production function. At this point, losses are zero and the actual output level equals the potential output. However, damage is inherent to livestock production. Input–output combinations are, therefore, located below the production function, excluding exceptional cases. At point $S_{0}$, there is a basic level of damage abatement without AMU. A graphical representation of the damage abatement function is shown in Figure 2, with the damage abatement effect on the y-axis and the use of damage-abatement inputs on the x-axis. In practice, the relationship between damage abatement and damage-abatement inputs is multidimensional because multiple damage-abatement inputs can be used to obtain the output. The damage abatement function only distinguishes the effect of AMU. Optimal AMU results in a shift of the input–output combination since an economically rational producer’s response is to increase productive input use and production intensity from point $S_{0}$ to point $S_{0}^{\prime }$ (see Figure 1).

The optimal damage abatement effect and the optimal level of damage-abatement input use are determined by the damage abatement function. The level of damage-abatement input use that generates optimal damage abatement is determined by the point where the function is tangent to the isoprofit line (see point $T_{0}$ in Figure 2). This involves a tangency condition in which the slope of the damage abatement function (i.e., the marginal product of damage-abatement input use) equals the slope of the corresponding isoprofit line. This point is found by taking the first derivative of π with respect to z, setting it equal to 0 and rewriting it in terms of ∂D/∂z (see Equations (7) and (8)).

(7) $$\frac{\partial \pi }{\partial z}=p\left(F\left(x\right)\times \frac{\partial D}{\partial z}\right)-w_{z}=0$$

(8) $$\frac{\partial D}{\partial z}=\frac{w_{z}}{p\cdot F\left(x\right)}$$

Equations (7) and (8) show that the marginal product of damage-abatement input use is determined by $w_{z}$, p and $F\left(x\right)$. A rational farmer uses more (less) damage-abatement inputs (i.e., AMs) when the price of damage-abatement inputs decreases (increases) and/or the price of the output increases (decreases), cet. par. Hence, the marginal product will increase (decrease) when $w_{z}$ decreases (increases), cet. par. In addition, this value will increase (decrease) when p increases (decreases), cet. par. The same effect applies to $F\left(x\right)$, cet. par. It is important to consider the substitutability of AMU, indicating that there are alternatives for antimicrobial agents as damage-abatement inputs.

The ${\mathrm{EV}}_{\mathrm{AMU}}$ is determined by comparing, for an individual farmer, the income obtained from production with a basic level of damage abatement without AMU (see production point $S_{0}$ in Figure 1) with the income obtained from production with optimal damage abatement including optimal AMU (see production point $S_{0}^{\prime }$ in Figure 1). These income levels are determined by the marginal product of productive input use and the marginal product of damage-abatement input use. Let Δy denote the change in output resulting from optimal AMU and Δx denote the change in productive input use resulting from optimal AMU, then the $EV_{AMU}$ is expressed as follows:

(9) $$EV_{AMU}=\Delta y\cdot p-\Delta x\cdot w_{x}-z\cdot w_{z}$$

The $EV_{AMU}$ of Equation (9) is equal to the change in individual producer surplus, which is obtained by comparing the level of production with and without optimal AMU (i.e., comparing points $S_{0}$ and $S_{0}^{\prime }$). The ${\mathrm{EV}}_{\mathrm{AMU}}$ is determined by both the marginal product of productive input use and the marginal product of damage-abatement input use. The ${\mathrm{EV}}_{\mathrm{AMU}}$ is negatively affected by $w_{x}$ and $w_{z}$, and positively affected by p, $F\left(x\right)$ and $D\left(z\right)$. In the short run, $F\left(x\right)$ and $D\left(z\right)$ are fixed, while p, $w_{x}$ and $w_{z}$ are variable. Hence, p, $w_{x}$ and $w_{z}$ determine the ${\mathrm{EV}}_{\mathrm{AMU}}$ in the short run. 

### 2.2. The Impact of Production System Improvements

**Figure 3:**
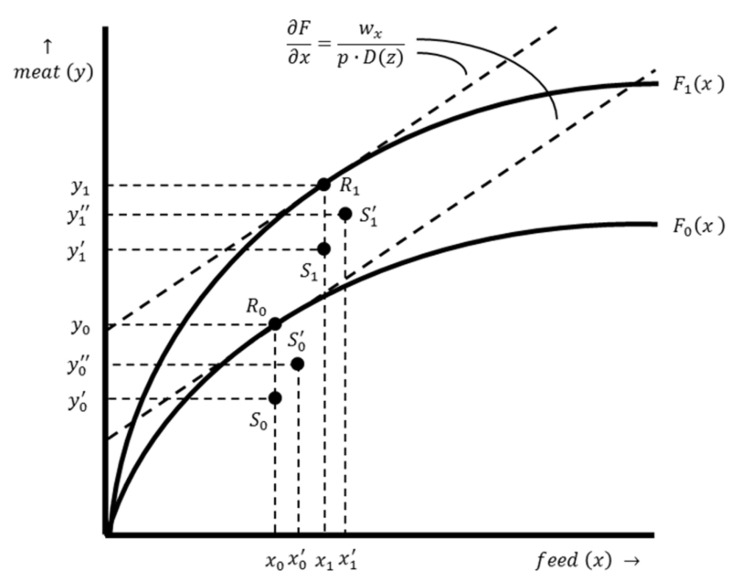
The production functions of a farm with a conventional production system and a farm with an improved production system. Production function $F_{0}\left(x\right)$ illustrates the relation between the output, meat $\left(y\right),$ and the productive input, feed $\left(x\right),$ for a farm with a conventional production system, while production function $F_{1}\left(x\right)$ illustrates the same input–output relation for a farm with an improved production system. The dashed lines represent isoprofit lines. The slope of these lines is $\frac{\partial F}{\partial x}=\frac{w_{x}}{p\cdot D\left(z\right)}$. Point $R_{0}$ shows the optimal input–output combination $\left(x_{0},y_{0}\right)$ for a farm with a conventional production system without damage, while point $R_{1}$ shows the optimal input–output combination $\left(x_{1},y_{1}\right)$ for a farm with an improved production system. Point $S_{0}$ shows the input–output combination $\left(x_{0},y_{0}^{,}\right)$ for a farm with a conventional production system in a situation with maximum damage and no AMU, while point $S_{1}$ shows the input–output combination $\left(x_{1},y_{1}^{\prime }\right)$ for a farm with an improved production system in the same situation. Point $S_{0}^{\prime }$ shows the input–out combination $\left(x_{0}^{\prime },y_{0}^{\prime \prime }\right)$ for a farm with a conventional production system resulting from optimal damage abatement, while point $S_{1}^{\prime }$ shows the input–out combination $\left(x_{1}^{\prime },y_{1}^{\prime \prime }\right)$ for a farm with an improved production system.

Successful and consistent implementation of preventive measures (e.g., biosecurity improvement) can reduce the prevalence and incidence of livestock diseases and mitigate their impact [28]. Production system improvements therefore reduce the need for AMU, but the production costs (either fixed or variable costs) are likely to increase at the same time. Such improvements increase the potential output, which results in an upward shift of the production function. This is shown in Figure 3, in which the production function $F_{0}\left(x\right)$ corresponds to a farm with a conventional production system and the production function $F_{1}\left(x\right)$ corresponds to a farm with an improved production system. The mathematical derivation of the optimal input–output combination is the same for both production systems (see Equations (5) and (6)). However, as illustrated in Figure 3, the optimal input–output combination for a farm with an improved production system $\left(x_{1},y_{1}\right)$ differs from the optimal input–output combination for a farm with a conventional production system $\left(x_{0},y_{0}\right)$.

**Figure 4:**
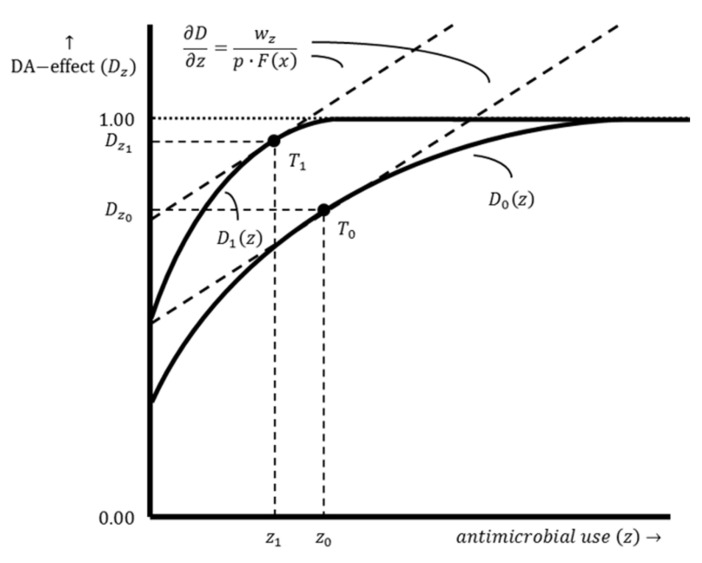
The damage abatement functions of a farm with a conventional production system and a farm with an improved production system. Damage abatement function $D_{0}\left(z\right)$ shows the relation between the damage abatement effect $\left(D_{z_{0}}\right)$ and the damage-abatement input use $\left(z_{0}\right)$ for a farm with a conventional production system, while damage abatement function $D_{1}\left(z\right)$ illustrates the relation between the damage abatement effect $\left(D_{z_{1}}\right)$ and the damage-abatement input use $\left(z_{1}\right)$ for a farm with an improved production system. The dashed lines represent isoprofit lines. The slope of these lines is $\frac{\partial D}{\partial z}=\frac{w_{z}}{p\cdot F\left(x\right)}$. Point $T_{0}$ shows the optimal damage abatement effect $\left(D_{z_{0}}\right)$ resulting from an optimal level of AMU $\left(z_{0}\right)$, while point $T_{1}$ shows the optimal damage abatement effect $\left(D_{z_{1}}\right)$ resulting from an optimal level of AMU $\left(z_{1}\right)$.

The likely impact of production system improvements on the use of damage-abatement inputs (i.e., AMs) is shown in Figure 4. Production system improvements are assumed to result in an outward shift of the damage abatement function since the level of damage abatement without AMU becomes higher. In addition, the damage abatement function is steeper since the maximum attainable damage abatement effect can be reached more quickly with AMU due to the production system improvements. The mathematical derivation of the optimal damage abatement effect and the optimal damage-abatement input use is the same for both production systems and, therefore, similar to Equations (7) and (8). However, as Figure 4 shows, the optimal damage abatement effect is higher for a farm with an improved production system $\left(D_{z_{1}}\right)$ compared to a farm with a conventional production system $\left(D_{z_{0}}\right)$, while the optimal level of damage-abatement input use (i.e., AMU) is lower for a farm with an improved production system $\left(z_{1}\right)$ compared to a farm with a conventional production system $\left(z_{0}\right)$.

The $EV_{AMU}$ in the situation with production system improvements is determined for two situations. First, the $EV_{AMU}$ is determined for a farm that has made investments in production system improvements by comparing the input–output combinations $S_{1}$ and $S_{1}^{\prime }$ (see Figure 3). The expression of the ${\mathrm{EV}}_{\mathrm{AMU}}$ in such a situation is, therefore, similar to Equation (9). Second, the $EV_{AMU}$ is different for a farmer with a conventional production system who considers investing in production system improvements. Assuming that investments have taken place, let Δy denote the change in output in the situation with and without production system investments; Δproduction costs denote the change in production costs associated with the investment; Δx denote the change in productive input use; and Δz denote the change in AMU. The $EV_{AMU}$ is expressed as follows:

(10) $$EV_{AMU}=\Delta y\cdot p-\Delta \mathrm{production}\;\mathrm{costs}-\Delta x\cdot w_{x}-\Delta z\cdot w_{z}$$

Equation (10) shows that a rational farmer will only invest in production system improvements when the $EV_{AMU}$ is positive (i.e., $\Delta production\;costs<\Delta y\cdot p-\Delta x\cdot w_{x}-\Delta z\cdot w_{z}$). Compared to the baseline model, an additional determinant of the $EV_{AMU}$ is the change in annual production costs resulting from investments in production system improvements.

### 2.3. The Impact of Risk and Risk Attitude

In the baseline model and the extended model with the impact of production system improvements, the impacts of risk and risk attitude are not taken into account. However, risk and uncertainty do affect the ${\mathrm{EV}}_{\mathrm{AMU}}$. In the von Neumann–Morgenstern utility theory [29], it is assumed that farmers aim to maximize the expected utility of income instead of income itself. Uncertainty enters through production risks, i.e., stochastic production (especially with respect to input–output quantities). The risk preferences of a farmer are implemented by assuming a mean–variance utility function, introduced by Markowitz [30], in which the certainty equivalent of a farmer is expressed in terms of the mean and the variance. This function assumes linear mean–variance risk preferences, which imply constant absolute risk aversion, i.e., the preferred option in a risky choice situation is unaffected by the addition or subtraction of a constant amount to all pay-offs [31]. Following Sargent [32], the farmer maximizes the mean profit minus the risk premium (i.e., the variance multiplied by a constant denoted as α). The larger α, the more risk averse the farmer. Hence, the utility of the farmer is in increasing the mean profit and decreasing the variance in profit. The more risk averse a farmer is, the higher the rate of decrease in utility with respect to the variance in profit. Let $\bar{\pi }$ denote the mean profit, α denote the measure of risk aversion, and $\sigma_{\pi }^{2}$ denote the variance of profit, then the expected utility of a farmer is expressed as follows:

(11) $$E\left(U\left(\pi \right)\right)=\bar{\pi }-\frac{1}{2}\alpha \cdot \sigma_{\pi }^{2}=\bar{p\left(F\left(x\right)\cdot D\left(z\right)\right)-w_{x}\cdot x-w_{z}\cdot z}-\frac{1}{2}\alpha \cdot \sigma_{\pi }^{2}$$

**Figure 5:**
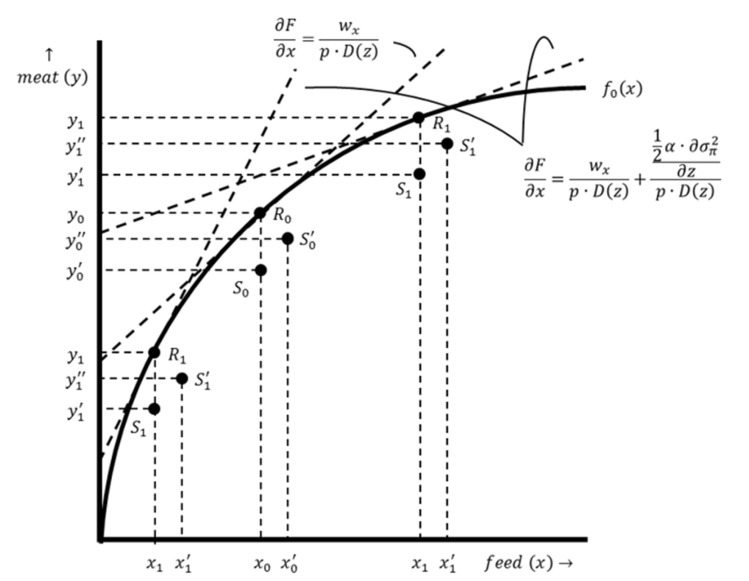
The optimal input–output combinations of risk-neutral and risk-averse farmers. Production function $F_{0}\left(x\right)$ illustrates the relation between the output, meat $\left(y\right),$ and the productive input, feed $\left(x\right),$ for a farm with a conventional production system. The dashed lines represent isoprofit lines; the slope of one of these lines is $\frac{\partial F}{\partial x}=\frac{w_{x}}{p\cdot D\left(z\right)}$, while the slope of the other isoprofit lines is $\frac{\partial F}{\partial x}=\frac{w_{x}}{p\cdot D\left(z\right)}+\frac{\frac{\frac{1}{2}\alpha \cdot \partial \sigma_{\pi }^{2}}{\partial x}}{p\cdot D\left(z\right)}$. Point $R_{0}$ shows the optimal input–output combination $\left(x_{0},y_{0}\right)$ for a risk-neutral farmer with a conventional production system, while point $R_{1}$ shows the optimal input–output combination $\left(x_{1},y_{1}\right)$ for a risk-averse farmer. Point $S_{0}$ shows the input–output combination $\left(x_{0},y_{0}^{\prime }\right)$ for a risk-neutral farmer in a situation with maximum damage and no AMU, while point $S_{1}$ shows the input–output combination $\left(x_{1},y_{1}^{\prime }\right)$ for a risk-averse farmer. Point $S_{0}^{\prime }$ shows the input–out combination $\left(x_{0}^{\prime },y_{0}^{\prime \prime }\right)$ for a risk-neutral farmer resulting from optimal damage abatement, while point $S_{1}^{\prime }$ shows the input–out combination $\left(x_{1}^{\prime },y_{1}^{\prime \prime }\right)$ for a risk-averse farmer.

According to the conventional profit maximization criterion, the optimal input–output combination is denoted at point $R_{0}$ in Figure 5. At this point, the production function is tangent to the isoprofit line. However, under utility maximization, the optimal input–output combination is determined by point $R_{1}$, where the production function is tangent to the iso-utility line (see Figure 5). This point is found by taking the first derivative of $U\left(\pi \right)$ with respect to x, setting it equal to 0 and rewriting it in terms of ∂F/∂x (see Equations (12) and (13)).

(12) $$\frac{\partial U\left(\pi \right)}{\partial x}=p\left(\frac{\partial F}{\partial x}\cdot D\left(z\right)\right)-w_{x}-\frac{\frac{1}{2}\alpha \cdot \partial \sigma_{\pi }^{2}}{\partial x}=0$$

(13) $$\frac{\partial F}{\partial x}=\frac{w_{x}}{p\cdot D\left(z\right)}+\frac{\frac{\frac{1}{2}\alpha \cdot \partial \sigma_{\pi }^{2}}{\partial x}}{p\cdot D\left(z\right)}$$

Equation (13) shows that the marginal product of productive input use is determined by $w_{x}$, p, $D\left(z\right)$, α and $\sigma_{\pi }^{2}$. The effects of $w_{x}$, p and $D\left(z\right)$ on the marginal product are similar to the standard profit maximization situation. As α is assumed to be positive, the more risk averse a farmer is, the lower the marginal product of productive input use, cet. par. The effect of $\sigma_{\pi }^{2}$ on productive input use can be positive (i.e., when a higher variance in profit results in higher productive input use) or negative (i.e., when a higher variance in profit results in lower productive inputs use), cet. par. Both effects are shown in Figure 5, where a positive (negative) effect results in a higher (lower) input–output combination compared to the initial situation. 

**Figure 6:**
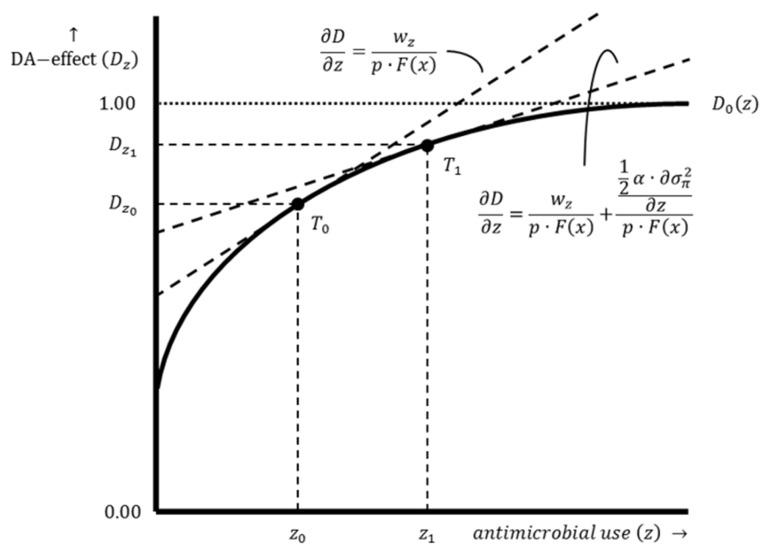
The optimal damage abatement effect of risk-neutral and risk-averse farmers. Damage abatement function $D_{0}\left(z\right)$ illustrates the relation between the damage abatement effect $\left(D_{z}\right)$ and the damage-abatement input use $\left(z\right)$ for a farm with a conventional production system. The dashed lines represent isoprofit lines. The slope of one of these lines is $\frac{\partial D}{\partial z}=\frac{w_{z}}{p\cdot F\left(x\right)}$. The slope of the other isoprofit line is $\frac{\partial D}{\partial z}=\frac{w_{z}}{p\cdot F\left(x\right)}+\frac{\frac{\frac{1}{2}\alpha \cdot \partial \sigma_{\pi }^{2}}{\partial z}}{p\cdot F\left(x\right)}$. Point $T_{0}$ shows the optimal damage abatement effect $\left(D_{z_{0}}\right)$ for a risk-neutral farmer, resulting from an optimal level of AMU $\left(z_{0}\right)$. Point $T_{1}$ shows the optimal damage abatement effect $\left(D_{z_{1}}\right)$ for a risk-averse farmer, resulting from an optimal level of AMU $\left(z_{1}\right)$.

The optimal damage abatement effect and the level of optimal damage-abatement input use are found at the point where the damage abatement function is tangent to the iso-utility line (see point $T_{1}$ in Figure 6). This point is found by taking the first derivative of $U\left(\pi \right)$ with respect to z, setting it equal to 0 and rewriting it in terms of ∂D/∂z (see Equations (14) and (15)).

(14) $$\frac{\partial U\left(\pi \right)}{\partial z}=p\left(\frac{\partial D}{\partial z}\cdot F\left(x\right)\right)-w_{z}-\frac{\frac{1}{2}\alpha \cdot \partial \sigma_{\pi }^{2}}{\partial z}=0$$

(15) $$\frac{\partial D}{\partial z}=\frac{w_{z}}{p\cdot F\left(x\right)}+\frac{\frac{\frac{1}{2}\alpha \cdot \partial \sigma_{\pi }^{2}}{\partial z}}{p\cdot F\left(x\right)}$$

The marginal product of damage-abatement input use is determined by $w_{z}$, p, $F\left(x\right)$, α and $\sigma_{\pi }^{2}$. The effects on the marginal product are similar to the standard profit maximization situation. As α is assumed to be positive, the more risk averse a farmer, the lower the marginal product of damage-abatement input use, cet. par. The effect of $\sigma_{\pi }^{2}$ on damage-abatement input use (i.e., AMU) can be positive (i.e., when a higher variance in profit results in more intensive AMU) or negative (i.e., when a higher variance in profit results in less intensive AMU), cet. par. However, since damage-abatement input use reduces damage, it is clear that damage-abatement input use (i.e., AMU) reduces $\sigma_{\pi }^{2}$. Therefore, the more risk averse a farmer, the more damage-abatement inputs are used to reduce $\sigma_{\pi }^{2}$. 

The $EV_{AMU}$ is obtained by extending Equation (9) with the change in α and $\sigma_{\pi }^{2}$:

(16) $${\mathrm{EV}}_{\mathrm{AMU}}=\Delta y\cdot p-z\cdot w_{z}-\Delta x\cdot w_{x}-\Delta \left(\frac{1}{2}\alpha \cdot \sigma_{\pi }^{2}\right)$$

The $EV_{AMU}$ is affected by the determinants p, $w_{x}$, $w_{z}$, $F\left(x\right)$ and $D\left(z\right)$. In the short run, $F\left(x\right)$ and $D\left(z\right)$ are fixed, while p, $w_{x}$ and $w_{z}$ are variable. Hence, p, $w_{x}$ and $w_{z}$ determine whether the $EV_{AMU}$ is positive in the short run. In addition, the $EV_{AMU}$ is affected by α and $\sigma_{\pi }^{2}$. The higher the risk aversion of a farmer (i.e., the higher the α), the higher the $EV_{AMU}$. Similarly, an increase in $\sigma_{\pi }^{2}$ also increases the $EV_{AMU}$.

## 3. Conclusions

The aim of this study was to develop a theoretical framework for assessing the $EV_{AMU}$ and to determine the factors that affect the $EV_{AMU}$. The proposed framework can be used to derive policy recommendations to reduce AMU in livestock production by influencing the $EV_{AMU}$. The framework shows that the $EV_{AMU}$ is negatively affected by the price of productive inputs and the price of damage-abatement inputs, and positively affected by the price of the output, the input–output combination and the damage abatement effect. In addition, the framework shows that the $EV_{AMU}$ is positively affected by the degree of risk aversion and the variance in profit. An understanding of the $EV_{AMU}$ and factors that affect the $EV_{AMU}$ can help identify pathways on how to reduce AMU in livestock production. 

Although the proposed theoretical framework includes a broad set of situations and factors that affect the $EV_{AMU}$ for individual livestock farmers, other situations (and factors) may also affect the $EV_{AMU}$. Furthermore, situations can be intertwined in reality. For example, the general perception of farmers is that the costs of non-antimicrobial alternatives and production system improvements are outweighed by the effect and costs of current AMU [16,33,34]. Hence, risk affects the decisions of individual farmers to invest in production system improvements. Komarek et al. [35] identified five major types of risk in agriculture that can play a role. 

This study provides the elements that affect the economic value of AMU, which provides insight into how to reduce AMU in livestock production. There are currently no global targets for reducing AMU and there is no understanding of how to set such targets. Hence, agreement is still needed about the level of AMU that is responsible and sustainable in the long run. A complete ban would have serious effects on animal health, animal welfare and productivity [36]. Adverse effects of a ban on AMU would be at least partially softened if cost-effective non-antimicrobial alternatives are available. However, such alternatives (including probiotics and prebiotics) are still experimental [37], and their efficacy is unclear and likely to be variable [36]. Hence, more research is urgently needed on the effects of reducing AMU and potential alternatives to AMU in livestock production. Although there is not yet a consensus about an acceptable level of AMU in livestock production from both an economic and a veterinary point of view, there is general agreement that AMU needs to be reduced. The theoretical framework presented in this study provides a solid theoretical basis for understanding the behavior of individual farmers with regard to AMU and, therefore, for conducting empirical studies to develop effective policies that can reduce AMU in livestock production. From an economic point of view, AMU is a source of negative externality. The total economic value of AMU in food-producing animals should integrate the economic losses due to AMR.

## Data Availability

Not applicable.
